# Induction of Hypoxic Response in Caco-2 Cells Promote the Expression of Genes Involved in SARS-CoV-2 Endocytosis and Transcytosis

**DOI:** 10.1134/S1607672922050118

**Published:** 2022-10-27

**Authors:** S. A. Nersisyan

**Affiliations:** 1 Faculty of Biology and Biotechnology, National Research University Higher School of Economics, Moscow, Russia; 2 Institute of Molecular Biology (IMB), National Academy of Sciences of the Republic of Armenia, Yerevan, Armenia

**Keywords:** Caco-2, intestine, hypoxia, oxyquinoline, SARS-CoV-2, ACE2, LDL, transcriptome, miRNA

## Abstract

In the present manuscript we analyzed the influence of hypoxic response in Caco-2 cells on the expression of genes and miRNAs involved in the mechanisms of intracellular transport of SARS-CoV-2 viral particles, especially endocytosis and transcytosis. With the use of RNA sequencing of Caco-2 cells treated with hypoxia-inducing oxyquinoline derivative, we showed two-fold increase in the expression of the main SARS-CoV-2 receptor ACE2. Expression of the non-canonical receptor TFRC was also elevated. We also observed a significant increase in the expression levels of genes from the low-density lipoprotein (LDL) receptor family, which play a crucial role in the transcytosis: *LDLR, LRP1, LRP4*, and *LRP5.* Upregulation of LDLR was coupled with the downregulation of hsa-miR-148a-3p, which can directly bind to LDLR mRNA. Thus, the hypoxic response in Caco-2 cells includes upregulation of genes involved in the mechanisms of endocytosis and transcytosis of SARS-CoV-2 viral particles.

Active replication of the SARS-CoV-2 virus in the intestine may be the cause of gastrointestinal symptoms in patients with COVID-19 [1]. It is known that, in the intestines of a significant proportion of those who have recovered from COVID-19, viral RNA is stored in the interval from one week to several months. Moreover, the long-term presence of viral RNA correlates with digestive disorders, which may be one of the factors of post-COVID syndrome [2, 3]. Hypoxia is one of the main inducers of intestinal pathologies, including inflammation and colorectal cancer [4]. The role of hypoxia in the interactions between SARS-CoV-2 and intestinal cells has not yet been established.

The main “entrance gate” into the cell for the SARS-CoV-2 virus particle is the ACE2 receptor, which is expressed on the surface of epithelial cells in many organs, including the lungs and intestines [5]. The further fate of the virus may include transcytosis, allowing the virus to cross the intestinal barrier, which may have important clinical implications. Previously, it was found that one of the most suitable cell models for studying endocytosis and transcytosis of SARS-CoV-2 viral particles are Caco-2 cells, which express all the necessary factors [6].

MicroRNAs are a class of short noncoding RNAs that downregulate gene expression. Binding of the microRNA seed region (nucleotides 2–7 from the 5' end of the molecule) to the 3'-untranslated region (3'-UTR) of the target mRNA leads to mRNA degradation or translation arrest [7, 8]. We have previously shown that the miR-200 miRNA family suppresses ACE2 expression [9]. The search for the regulatory mechanisms for other genes involved in the interactions between SARS-CoV-2 and cells is of great inte-rest.

In this work, using next-generation sequencing, we analyzed the expression profile of genes and miRNAs of differentiated Caco-2 cells under the influence of an oxyquinoline derivative, which is an inhibitor of HIF prolyl hydroxylase and mimics hypoxia by stabilizing HIF1A, the main transcription factor induced by hypoxia [10].

The experiments were performed as described in [11]. Briefly, Caco-2 cells were obtained from the Russian Cell Culture Collection (Institute of Cytology of the Russian Academy of Sciences, St. Petersburg, Russia) and incubated for 21 days under differentiation conditions. The cells were exposed to the oxyquinoline derivative 4896–3212 (Research Institute of Chemical Diversity, Khimki, Russia), 7-((4-(tert-butyl)phenyl)((4-methylpyridin-2-yl)amino)methyl)qui- noline-8-ol (for details, see [12]), at a concentration of 5 μM. After 24 h of incubation, the cells were lysed for further analysis. Three biological replicates were used in each group (exposure and control). RNA was isolated using the Qiagen miRNeasy Mini Kit (Qiagen, Hilden, Germany). Libraries for mRNA and microRNA sequencing were prepared using the Illumina Stranded mRNA Library Prep Kit and the NEBNext Multiplex Small RNA Library Prep Kit for Illumina (Illumina, San Diego, United States), respectively. An Illumina NextSeq 550 sequencer was used.

The quality of the original FASTQ sequencing files was assessed using the FastQC software version 0.11.9 (Babraham Bioinformatics, Cambridge, England), read adapters were trimmed using cutadapt version 2.10. Unnormalized mRNA and miRNA expression tables were generated by mapping sequencing reads with STAR version 2.7.5b and miRDeep2 version 2.0.1.2, respectively. The resulting tables were normalized and filtered using edgeR version 3.30.3, yielding fragments per kilobase of transcript per million mapped reads (FPKM) and reads per million mapped reads (RPM) scales for mRNA and miRNA sequencings, log base 2, respectively. Analysis of the differential expression of mRNA and miRNA was performed using DESeq2 version 1.28.1. Changes in expression with a fold change of at least 1.5 and a false discovery rate (FDR) < 0.05 were considered significant (FDR was calculated using the Benjamini–Hochberg procedure).

Validation of changes in the expression of key mRNAs was performed using real-time polymerase chain reaction with reverse transcription (RT-PCR) as described in [13]. The sequences of primers used in experiments are shown in Table 1. Differential expression according to real-time PCR data was analyzed using the ΔΔCt method and Student’s *t* test. *ACTB* was used as a reference gene.

**Table 1.** Primers used in real-time PCR and their efficiency

Gene	Forward primer	Reverse primer	Efficiency
*ACE2*	AGAGAAGTGGAGGTGGATGGTCTTTT	GCGGGGTCACAGTATGTTTCATCA	2.10
*TFRC*	GTCCAGACAATCTCCAGAGCTGC	TCTGTTTTCCAGTCAGAGGGACAGT	1.97
*LDLR*	CATTGTCTCCCCATCGTGCTC	AGCTGTAGCCGTCCTGGTTG	2.06
*LRP1*	GGCGTCACTTGCTTGGCGAA	TGAATCGGTCCGAGGGGCAG	2.08
*LRP4*	GGGAGTGTGAGGAGGACGAGT	TGGCACTGCTGAGGGACAGTTC	1.98
*LRP5*	TGCGATGACCAGAGCGACGA	GCAGGCAGATGGCGTCACAG	1.97
*ACTB*	CTGGAACGGTGAAGGTGACA	AAGGGACTTCCTGTAACAACGCA	2.03

The genes involved in the processes of endocytosis and transcytosis were taken from the Gene Ontology (GO) database. Functionality enrichment analysis was performed with the use of the DAVID web service version 2021 using the Kyoto Encyclopedia of Genes and Genomes (KEGG) biological pathway database. MicroRNA targets were predicted using the miRDB web portal version 6.0.

As a result of comparison of mRNA expression profiles between the samples with induced hypoxia and the control cells, 6309 differentially expressed genes were found. Analysis of the enrichment of the differentially expressed genes by functional affiliation revealed a distinct activation of the HIF1A signaling pathway (KEGG hsa04066, FDR < 0.05) and anaerobic glycolysis (KEGG hsa00010, FDR < 0.05), which is evidence of the induction of a hypoxic response when Caco-2 cells were treated with oxyquinoline. The same analysis did not reveal activation of pathways associated with possible toxic effects on cells (in particular, apoptosis and DNA repair). A twofold increase in the expression of the *ACE2* gene (FDR < 0.05), which encodes the main receptor for the SARS-CoV-2 virus, was observed (Fig. 1). In addition to the canonical receptor, a twofold increase in the expression of mRNA of the *TFRC* transferrin 1 receptor, which is also capable of binding the SARS-CoV-2 S protein with subsequent endocytosis of the viral particle, was detected [14].

**Fig. 1.**
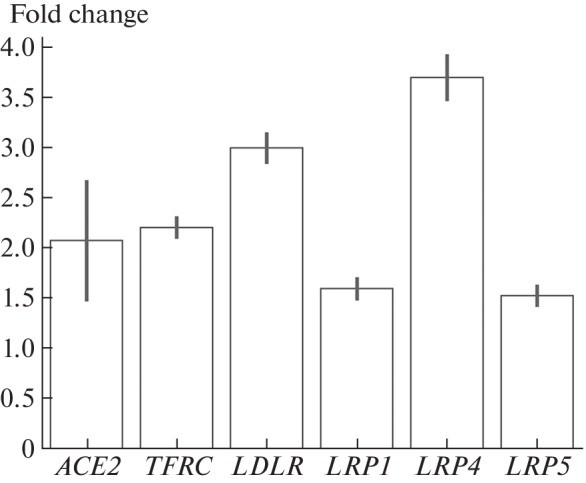
Fold changes in expression levels of *ACE2*, *TFRC*, *LDLR*, *LRP1*, *LRP4*, and *LRP5* genes during hypoxic response in Caco-2 cells. The vertical bars show the standard deviations calculated with DESeq2 using three experimental and three control samples (FDR < 0.05 for all genes).

When analyzing the differentially expressed genes encoding the proteins involved in endocytosis and transcytosis, a statistically significant increase in the expression level of the genes for the low-density lipoprotein receptor (LDL) family, including *LDLR* (fold change 3.0), *LRP1* (fold change 1.6), *LRP4* (fold change 3.7), and *LRP5* (fold change 1.5) (see Fig. 1). One of the well-studied functions of these receptors is LDL transcytosis through various barriers [15, 16]. It was previously shown that an increased expression of the LDLR receptor is a severe course factor in patients with COVID-19 [17, 18]. The changes in the expression of *ACE2*, *TFRC*, *LDLR*, *LRP1*, *LRP4*, and *LRP5* genes were confirmed by real-time PCR: a statistically significant increase in expression levels (*p* < 0.05) was observed for all genes (the fold change in expression, according to real-time PCR data, was 1–1.7 times higher than according to RNA sequencing data).

To search for the possible causes for the changes in gene expression at hypoxic response, we analyzed the differentially expressed microRNAs whose expression levels were assessed by sequencing. Two highly expressed microRNAs with a significant (FDR < 0.05) difference in expression between oxyquinoline-treated and control cells were hsa-miR-210-3p and hsa-miR-148a-3p. The expression of hsa-miR-210-3p increased 1.7 times under the influence of oxyquinoline, which is another evidence of successful induction of the hypoxic response: increased expression of hsa-miR-210-3p is a generally accepted marker of the cellular response to hypoxia [19].

The next microRNA, hsa-miR-148a-3p, ranked fourth in the absolute expression level among all microRNAs of the control Caco-2 cells, accounting for 7% of all sequencing reads. A 1.5-fold decrease in the hsa-miR-148a-3p microRNA expression level as a result of the treatment with oxyquinoline was a possible cause for the increase in the *LDLR* gene expression. Namely, the 3′-untranslated region of *LDLR* mRNA contained two binding sites for the seed regions of hsa-miR-148a-3p miRNA of types 7mer-m8 (complementarity of nucleotides 2–8 from the 5' end of miRNA) and 8mer (7mer-m8, as well as adenine is located opposite the first nucleotide from the 5' end of the microRNA) (see Fig. 2). Interaction of hsa-miR-148a-3p and *LDLR* was confirmed previously using luciferase reporter constructs [20].

**Fig. 2.**
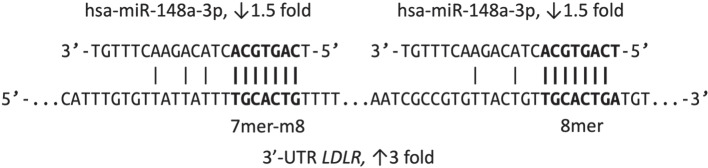
Binding sites for hsa-miR-148a-3p in the 3'-untranslated region of *LDLR* mRNA. The expanded miRNA seed regions and the corresponding sites in the mRNA targets are shown in bold.

Thus, it was shown that the simulation of hypoxia with an oxyquinoline derivative in Caco-2 cells is accompanied by an increased expression of *ACE2* and *TFRC* genes, encoding receptors capable of binding to the S-protein of the SARS-CoV-2 virus, as well as an increased expression of genes for the receptors of the LDL family, which are involved in the mechanisms of endocytosis and transcytosis. One of the causes for the increase in the *LDLR* gene expression might be a decrease in the expression level of hsa-miR-148a-3p miRNA, which can directly bind to *LDLR* mRNA. Therefore, intestinal hypoxia may be an unfavorable factor in COVID-19.

*Abbreviations:* RT-PCR, real-time polymerase chain reaction, GO, gene ontology, FDR, false discovery rate, FPKM, fragments per kilobase of transcripts per million mapped, HIF, hypoxia-inducible factor, KEGG, Kyoto Encyclopedia of Genes and Genomes, RPM, reads per million mapped reads.
